# The Evolving Role of Artificial Intelligence in Dermatology: A Meta-Analysis of Diagnostic Performance, Clinical Applications, and Implementation Challenges (2003–2025)

**DOI:** 10.3390/diagnostics16172797

**Published:** 2026-08-31

**Authors:** Nina Ivanovic, Marius Florentin Popa, Ana-Olivia Toma, Nicolae Ciprian Pilut, Roxana Manuela Fericean, Daniela Crainic, Andreea Nelson Twakor, Daniela Vasilica Serban, Kersztin Lorett Csiki, Raluca Dumache

**Affiliations:** 1 Department of Dermatology, “Victor Babes” University of Medicine and Pharmacy Timisoara, Eftimie Murgu Square 2, 300041 Timisoara, Romania; nina.ivanovic@umft.ro (N.I.); pilut.nicolae@umft.ro (N.C.P.); manuela.fericean@umft.ro (R.M.F.); daniela.crainic@umft.ro (D.C.); daniela.serban@umft.ro (D.V.S.); 2 Doctoral School of Medicine “Victor Babes”, “Victor Babes” University of Medicine and Pharmacy Timisoara, Eftimie Murgu Square 2, 300041 Timisoara, Romania; kersztin.csiki@umft.ro; 3 Center for the Morphologic Study of the Skin (MORPHODERM), “Victor Babes” University of Medicine and Pharmacy Timisoara, Eftimie Murgu Square 2, 300041 Timisoara, Romania; 4 Research Center for Ethics in Human Genetic Identification (EtIGen), “Victor Babes” University of Medicine and Pharmacy Timisoara, Eftimie Murgu Square 2, 300041 Timisoara, Romania; raluca.dumache@umft.ro; 5 Department of Forensic Pathology, Faculty of Medicine, “Ovidius” University, Aleea Universitatii Nr 1, 900591 Constanta, Romania; marius.popa@univ-ovidius.ro; 6 Internal Medicine Department, Sf. Apostol Andrei” Emergency County Hospital, 145 Tomis Blvd., 900591 Constanta, Romania; 7 Department of Forensic Medicine, Bioethics, Medical Ethics and Medical Law, “Victor Babes” University of Medicine and Pharmacy Timisoara, Eftimie Murgu Square 2, 300041 Timisoara, Romania

**Keywords:** artificial intelligence, dermatology, melanoma, deep learning, convolutional neural network, systematic review, meta-analysis, diagnostic performance, algorithmic bias

## Abstract

**Background:** Artificial intelligence (AI) has emerged as a transformative technology across dermatological practice, from automated lesion classification to whole-slide pathology analysis. Despite rapid growth in primary studies, a comprehensive synthesis of diagnostic performance, application breadth, and real-world implementation remains lacking. **Methods:** We conducted a PRISMA systematic review and meta-analysis of studies published from January 2000 to March 2025. We searched the PubMed, Cochrane, and ScienceDirect databases for studies reporting AI diagnostic performance in dermatology. **Results:** Of 30 included studies (28 valid after exclusion of two retracted publications), 60% focused on melanoma and related lesions. AI diagnostic performance improved markedly over five identified temporal eras (2003–2025), with a pooled AUROC of 0.92 (95% CI 0.87–0.96), Reitsma sensitivity of 0.88 (0.82–0.93), and Reitsma specificity of 0.85 (0.75–0.91). AI matched or surpassed specialist dermatologists in 71% of direct comparisons. Three randomized controlled trials (RCTs) were identified, with heterogeneous findings across different clinical applications: AI assistance significantly improved non-expert diagnostic accuracy in one trial (53.9% vs. 43.8%; *p* = 0.019), significantly reduced acne severity via personalized treatment recommendations in a second, and showed non-inferior diagnostic performance, but was not cost-effective in the third. The sole cost-effectiveness analysis found AI-assisted surveillance not cost-effective over a 2-year horizon. **Conclusions:** AI achieves dermatologist-level diagnostic accuracy in controlled settings; however, real-world evidence, algorithmic equity across skin phototypes, and health economic viability remain critical unresolved challenges. Prospective validation, mandatory demographic subgroup reporting, and cost-effectiveness modeling are essential prerequisites for safe and equitable clinical implementation.

## 1. Introduction

Skin diseases represent one of the most prevalent categories of human illness worldwide. According to the Global Burden of Disease 2010 study, skin conditions constitute the fourth-leading cause of non-fatal disease burden globally, affecting populations across all 187 countries surveyed [1]. Among skin malignancies, cutaneous melanoma poses the greatest threat to life: GLOBOCAN 2020 data estimate approximately 325,000 new cases and 57,000 deaths annually worldwide, with incidence projected to increase by over 50% by 2040, imposing a substantial and growing burden on healthcare systems in high-incidence regions including Western Europe and Australia [2]. Despite this epidemiological urgency, access to specialist dermatological care remains critically unequal. Geographic maldistribution of the dermatology workforce creates significant disparities in timely diagnosis, particularly in rural and underserved areas [2], where diagnostic delay is directly associated with advanced-stage presentation and poorer survival outcomes. Melanoma itself is not a single clinical or biological entity. Its four principal subtypes (superficial spreading melanoma, nodular melanoma, melanoma in situ, and acral lentiginous melanoma) differ substantially in anatomical distribution, growth pattern, and diagnostic difficulty. Acral lentiginous melanoma disproportionately affects patients with darker skin phototypes and poses distinct dermoscopic and clinical diagnostic challenges [3]. This clinical and technological heterogeneity is directly relevant to the AI equity concerns addressed later in this review, since diagnostic algorithms trained predominantly on non-acral, lighter-skin dermoscopic images may specifically underperform on acral lesions in patients with darker-skin phototypes.

The rapid maturation of AI and deep learning technologies has opened a transformative pathway for addressing these challenges across medicine broadly [2]. Dermatology, a fundamentally image-driven specialty, emerged as one of the earliest and most fertile domains for AI application. The rapid evolution of artificial intelligence has not been limited to diagnostic imaging, but has also extended into adjacent medical disciplines, including forensic medicine, where intelligent materials and AI-assisted technologies have demonstrated promising applications for human identification and personalized forensic investigations, highlighting the broad translational potential of AI across healthcare [4]. In 2017, Esteva et al. demonstrated that a single CNN trained on 129,450 clinical images could classify skin cancer at the level of board-certified dermatologists across 2032 disease categories, a landmark result that galvanized the field [5]. This was subsequently corroborated in a large prospective European reader study by Haenssle et al. in 2018, in which a CNN outperformed 58 international dermatologists in dermoscopic melanoma recognition on both sensitivity and specificity [6].

Since these foundational studies, the scope of AI in dermatology has expanded considerably beyond binary melanoma classification. Contemporary applications encompass teledermatology-integrated decision support, whole-slide-image pathology analysis, total-body photography surveillance, multimodal diagnostic systems, generative AI for patient education, and machine learning-driven treatment recommendation, spanning the full clinical pathway from prevention to precision oncology. Tschandl et al. demonstrated that human–computer collaboration across varying levels of clinical expertise and diverse teledermatology workflows could substantially improve diagnostic accuracy, establishing the “augmented intelligence” paradigm as the operational model for AI deployment in skin disease [7]. Despite this proliferation of published studies, systematic synthesis of this heterogeneous evidence base remains limited. A recent systematic review and meta-analysis by Krakowski et al. conducted in 2024 highlighted that real-world evidence for AI benefit in integrated human–AI clinical workflows remains sparse [8].

The present systematic review and meta-analysis therefore aims to provide a comprehensive, temporally structured synthesis of AI applications across dermatology from 2003 to 2025, evaluating diagnostic performance, clinical utility, technological evolution, and equity considerations, to inform evidence-based deployment of AI in dermatological practice.

## 2. Materials and Methods

### 2.1. Study Design

This study was conducted as a meta-analysis in accordance with the Preferred Reporting Items for Systematic Reviews and Meta-Analyses (PRISMA) 2020 guidelines [9]. The protocol was designed a priori to evaluate the development, validation, and clinical application of AI technologies across the spectrum of dermatological practice, with particular emphasis on diagnostic performance, clinical utility, temporal evolution of AI technologies, and equity in AI deployment. The review is registered in PROSPERO—CRD420261416910.

### 2.2. Eligibility Criteria

Studies were considered eligible if they met all predefined inclusion criteria and none of the exclusion criteria, as outlined in Table 1. Eligible studies were required to be original peer-reviewed research reporting quantitative outcomes for AI or machine learning systems applied to human dermatological conditions. No restriction was placed on AI technology type, disease category, or study design, in order to capture the full breadth of AI application in dermatology from early neural networks to contemporary large language models. Publications in languages other than English were excluded due to translation resource limitations.

### 2.3. Information Sources and Search Strategy

A systematic literature search was conducted across the PubMed, Cochrane Library, and ScienceDirect databases. The search covered publications from January 2000 to March 2025. No lower date limit was applied because the earliest eligible AI dermatology publications date to the late 1990s/early 2000s and excluding these would have precluded analysis of the historical evolution of AI technology in this field.

Search queries were constructed using medical subject headings (MeSH) [10] terms in PubMed. The core search strategy comprised three conceptual blocks combined with Boolean [11] AND: (1) AI/machine learning technology terms [artificial intelligence OR machine learning OR deep learning OR neural network OR convolutional neural network OR computer vision OR natural language processing OR large language model]; (2) dermatology domain terms [dermatology OR dermatological OR skin OR melanoma OR dermoscopy OR dermatoscopy OR cutaneous OR onychomycosis OR psoriasis OR eczema OR acne OR vitiligo]; and (3) outcome/application terms [diagnosis OR classification OR detection OR screening OR prediction OR treatment OR pathology OR histopathology OR teledermatology].

### 2.4. Study Selection Process

Following duplicate removal, two independent reviewers screened titles and abstracts against the predefined eligibility criteria. Disagreements at the title/abstract stage were resolved by discussion, and where necessary referral to a third reviewer. Full-text articles were retrieved for all records meeting eligibility criteria at the screening stage, as well as for all records where eligibility could not be determined from abstract information alone.

Full-text eligibility assessment was performed independently by two reviewers. Each article was evaluated against all inclusion and exclusion criteria. Reasons for full-text exclusion were recorded for each excluded article and are reported in the PRISMA flow diagram. Two articles were identified as formally retracted. These articles were retained in the study characteristics table for transparency, but excluded from all quantitative analyses and performance metric syntheses, reducing the analytic cohort to 28 studies. The full search export is in Supplementary Material S1.

**Figure 1:**
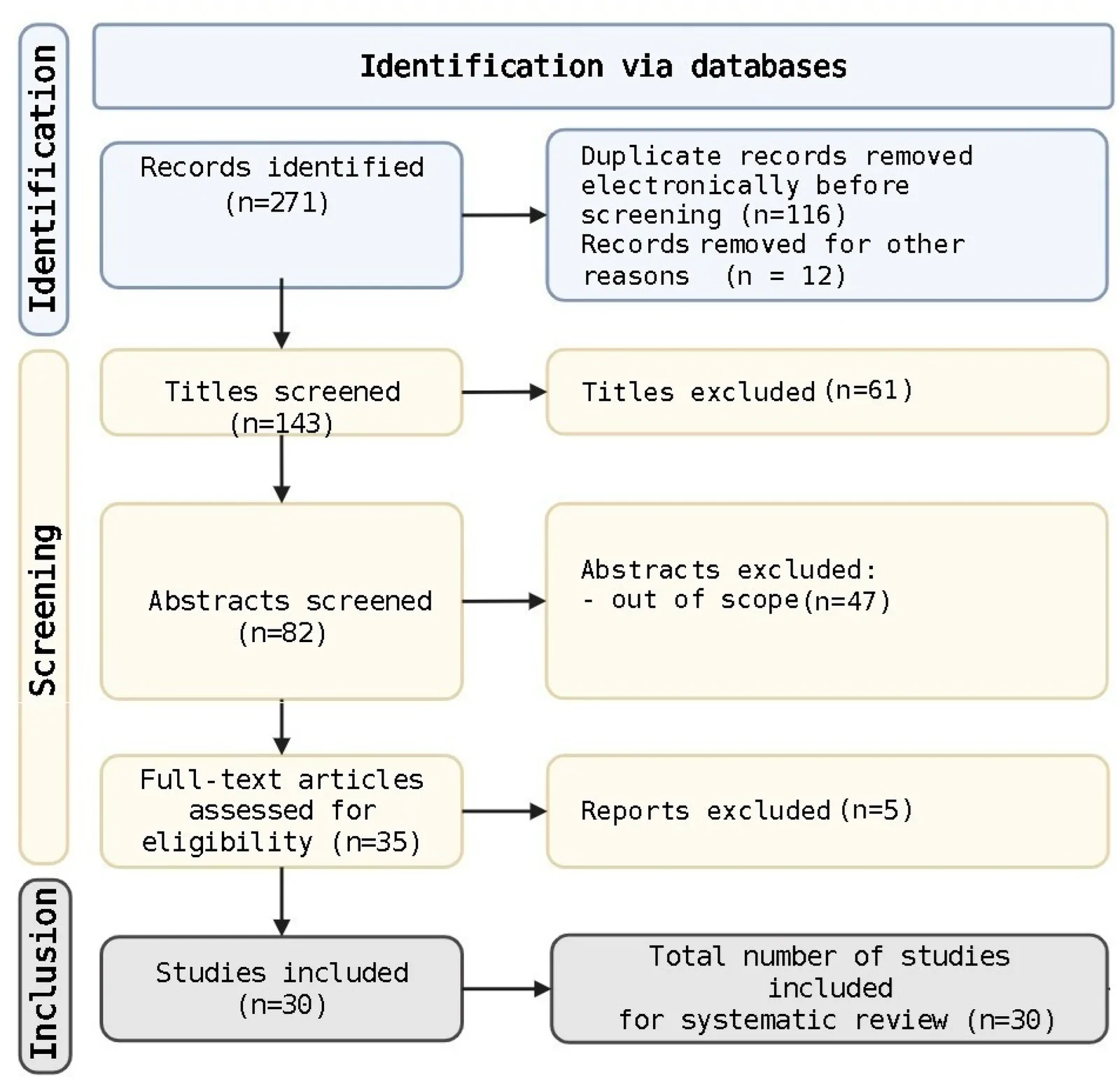
PRISMA flow diagram. Created with BioRender. Nelson Twakor, A. (2026). https://BioRender.com/vcmup9b (accessed on 25 August 2026).

The PRISMA study selection flow is illustrated in the PRISMA 2020 flow diagram (Figure 1).

### 2.5. Data Extraction

**Figure 2:**
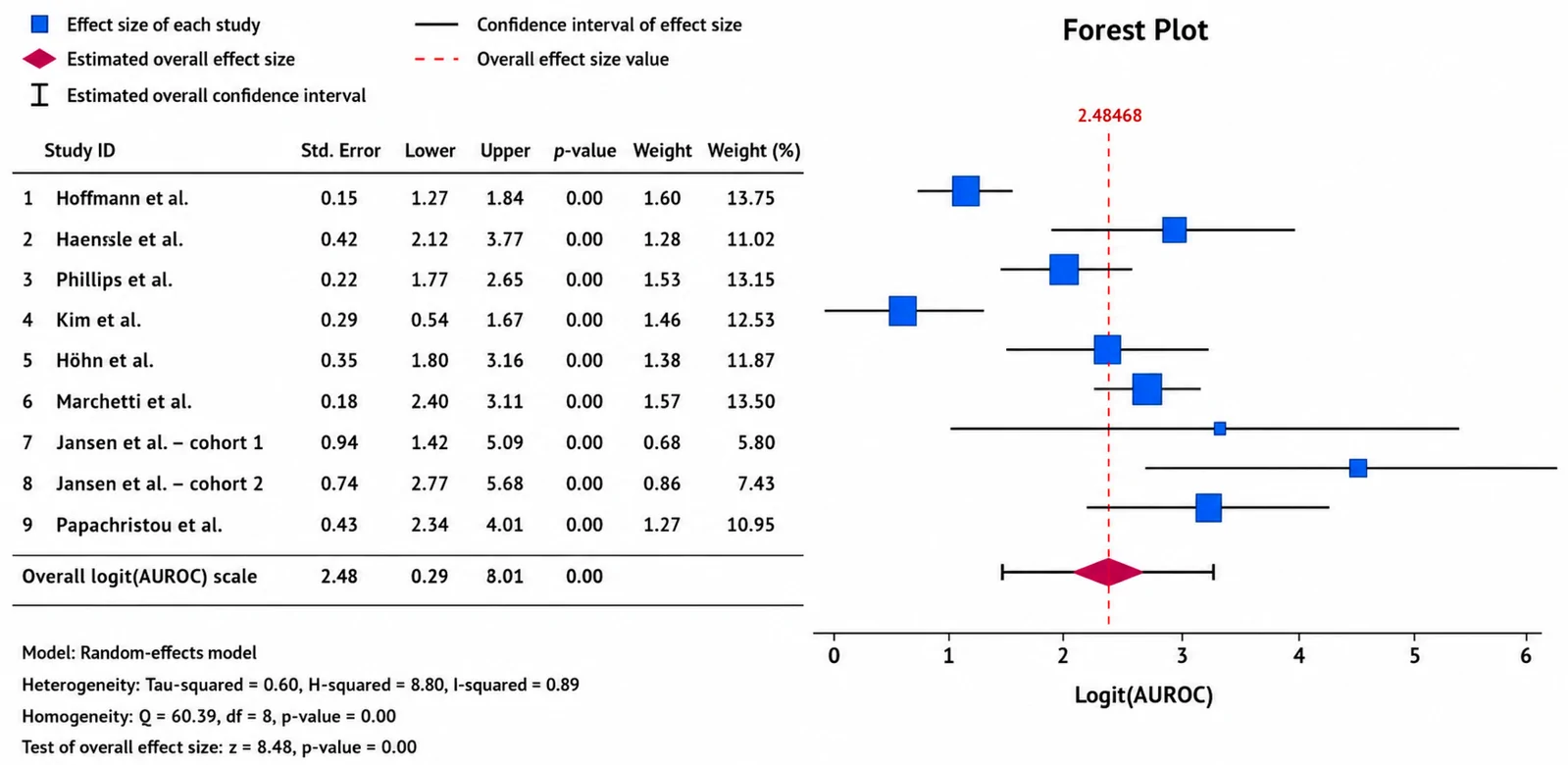
Forest plot of logit(AUROC) from the random-effect REML model (SPSS vs. 30.0). The pooled logit of 2.48 (1.91–3.06) corresponds to a back-transformed AUROC of 0.92 (0.87–0.96). Studies included Hoffmann et al. [46]; Haenssle et al. [22]; Phillips et al. [28]; Kim et al. [42]; Höhn et al. [41]; Marchetti et al. [37]; Jansen et al. [31]; Papachristou et al. [18].

**Figure 3:**
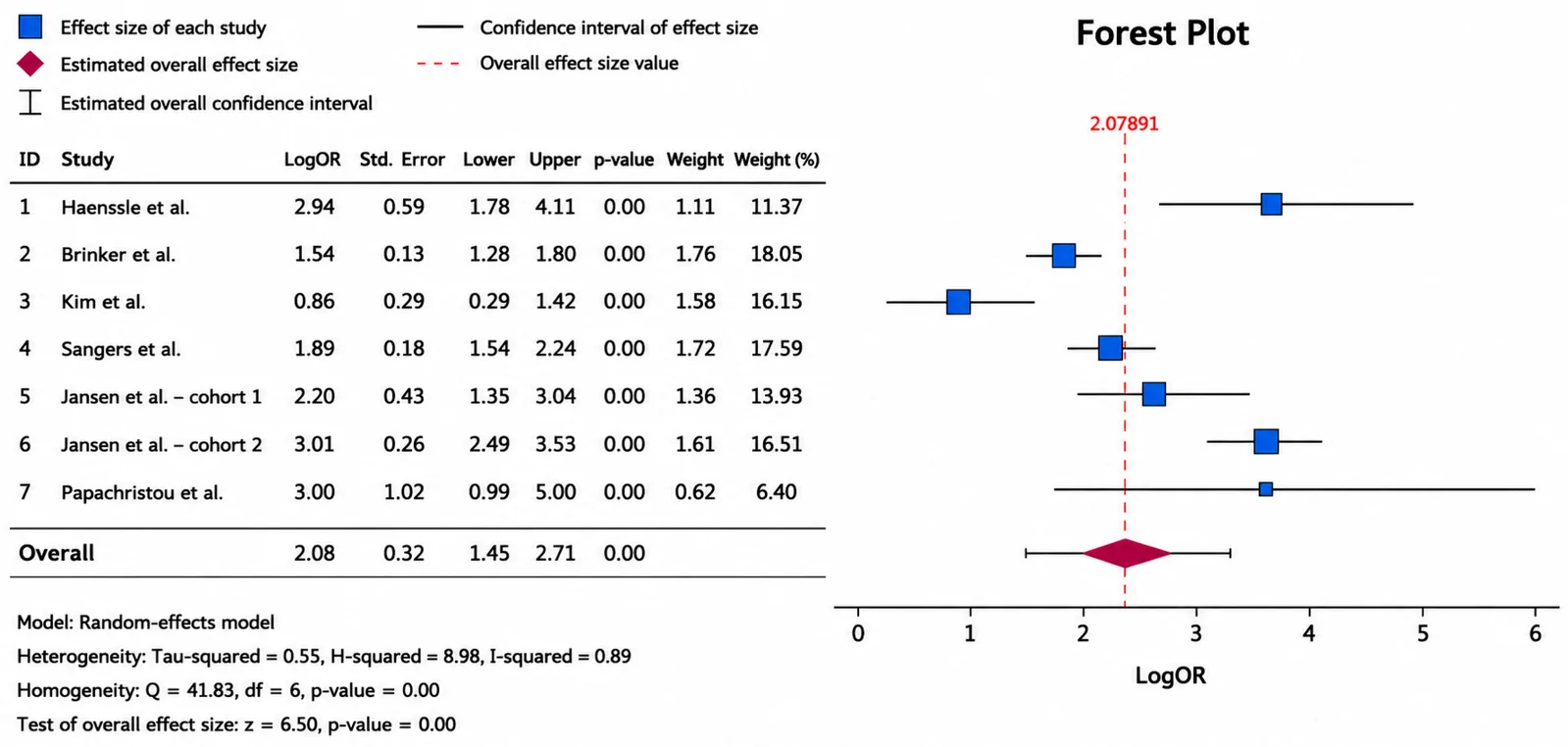
Forest plot of logit(sensitivity) from the random-effect REML model. The pooled logit of 2.08 (1.45–2.71) corresponds to a univariate sensitivity of 0.89 (0.81–0.94). The primary pooled estimate is the Reitsma sensitivity of 0.88 (0.82–0.93). Studies included Haenssle et al. [22]; Brinker et al. [36]; Kim et al. [42]; Sangers et al. [34]; Jansen et al. [31]; Papachristou et al. [18].

**Figure 4:**
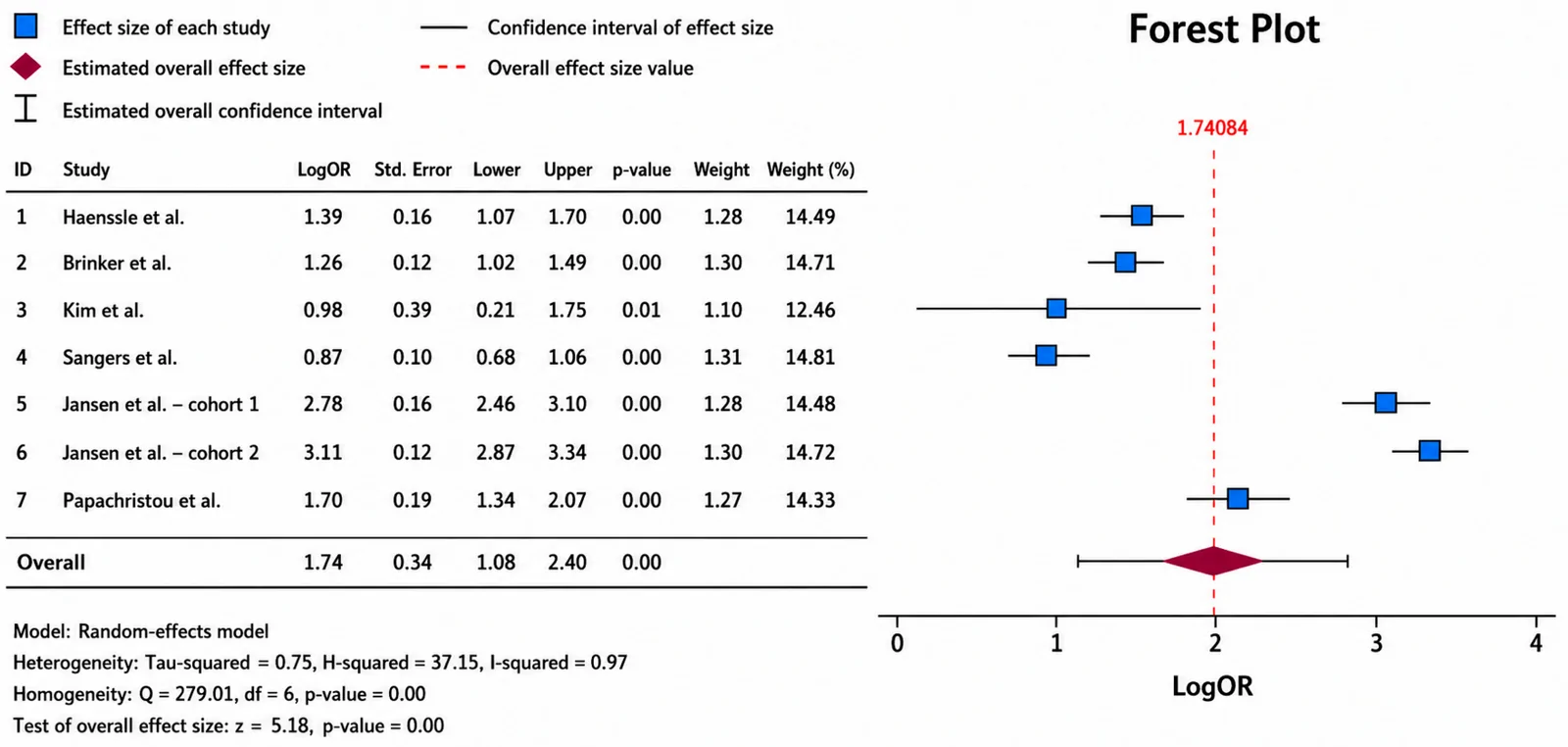
Forest plot of logit(specificity) from the random-effect REML model (SPSS vs. 30.0). The pooled logit of 1.74 (1.08–2.40) corresponds to a univariate specificity of 0.85 (0.75–0.92). The primary pooled estimate is the Reitsma specificity of 0.85 (0.75–0.91). Studies included Haenssle et al. [22]; Brinker et al. [36]; Kim et al. [42]; Sangers et al. [34]; Jansen et al. [31]; Papachristou et al. [18].

For studies reporting performance metrics at multiple operating thresholds, the metric corresponding to the primary analysis or the threshold yielding the closest sensitivity–specificity balance was extracted. For studies presenting multiple AI algorithms or model iterations, the estimate corresponding to the algorithm designated by the original authors as their primary or final model was extracted for the main pooled analysis. For studies with multiple cohorts or subgroups, a single prespecified primary effect estimate per study was extracted and used in the main pooled analysis, so that each study contributed exactly one estimate and no clustering adjustment was required. Study-level AUROC values, 2 × 2 counts, and source locators used for Figure 2, Figure 3 and Figure 4 are in Supplementary Material S2.

### 2.6. Risk-of-Bias Assessment

Risk of bias for included diagnostic accuracy studies was appraised qualitatively by two independent reviewers, informed by the domains of the Quality Assessment of Diagnostic Accuracy Studies-2 (QUADAS-2) tool and by AI-specific methodological concerns raised in the DECIDE-AI and TRIPOD + AI reporting frameworks [12], including provenance and separation of training–testing datasets, reporting of demographic subgroups, and independence of the external validation cohort. Disagreements between the two reviewers were resolved through consensus discussion. A formal domain-by-domain scoring system and a quantitative inter-rater agreement statistic were not applied and are noted as a limitation.

For interventional studies, risk of bias was assessed using the Revised Cochrane Risk of Bias Tool for Randomized Trials (RoB 2), evaluating domains of randomization process, deviations from intended interventions, missing outcome data, measurement of outcomes, and selection of reported results [13]. Risk-of-bias assessments were performed independently by two reviewers. Disagreements were resolved by consensus.

### 2.7. Statistical Analysis

#### 2.7.1. Software and Primary Meta-Analytic Outcome

Univariate random-effect models and the forest plots in Figure 2, Figure 3 and Figure 4 were generated in IBM SPSS Statistics version 30.0 (IBM Corp., Armonk, NY, USA) [14]. The primary bivariate analysis of sensitivity and specificity used the Reitsma random-effect model, fitted in Python 3 (SciPy) on the logit scale. The primary discrimination outcome was the area under the receiver operating characteristic curve (AUC/AUROC). Study-level AUROC values were transformed to the logit scale, logit(AUC) = ln[AUC/(1 − AUC)]. Standard errors on the logit scale were obtained from published 95% CIs when available, or by the delta method from a Hanley–McNeil standard error when only the AUROC and the numbers of diseased and non-diseased observations were reported. Pooling used a random-effect REML model on the logit scale. Only the pooled estimate and its 95% CI were back-transformed as AUC = 1/(1 + e^(−logit)^) so that the reported interval was constrained to (0, 1).

#### 2.7.2. Meta-Analytic Model Selection

Given the substantial methodological and clinical heterogeneity anticipated across included studies, a random-effect model was prespecified as the primary meta-analytic model [15]. This model assumes that individual-study effect sizes are drawn from a distribution of true effects and estimate between-study variance (τ^2^). Fixed-effect modeling was not used as the primary approach, as the assumption of a single common true effect size is implausible given the heterogeneity of AI technologies and clinical contexts represented in this corpus [16].

Sensitivity and specificity were not treated as independent primary outcomes. Studies that provided a reconstructible binary 2 × 2 table at a free operating point (k = 7 independent testing sets) were analyzed with the Reitsma bivariate random-effect model, which jointly estimates logit-sensitivity, logit-specificity, and their between-study correlation. SPSS Figure 3 and Figure 4 display the corresponding univariate logit models for visual inspection. The manuscript primary estimates are the Reitsma summaries. Only one independent testing set per study was included Multiclass top-k accuracy, fixed-sensitivity equity analyses, lesion-detection rates, and duplicate lesion sets were excluded from the quantitative pool and retained in the narrative synthesis. A continuity correction of 0.5 was reserved for any zero cell. None was required in the primary 2 × 2 tables.

#### 2.7.3. Heterogeneity Assessment

Between-study variability in pooled AUC, sensitivity, and specificity estimates was assessed through visual inspection of the forest plots and by comparing effect estimates across six prespecified moderator variables: AI technology category (CNN/deep learning vs. classical neural network vs. mobile app vs. 3D TBP system vs. multimodal DL vs. foundation model), skin condition (melanoma vs. non-melanoma conditions), study design (RCT/prospective trial vs. retrospective/model development study vs. diagnostic accuracy/reader study), clinical setting (specialist secondary/tertiary care vs. primary care/teledermatology vs. digital pathology laboratory), publication era (2000–2009 vs. 2010–2019 vs. 2020–2025), and comparator type (AI vs. specialist dermatologist vs. AI vs. non-specialist clinician vs. standalone AI). Between-study heterogeneity for the SPSS forest plots is reported as Cochran’s Q, I^2^, and τ^2^. For the Reitsma model, between-study standard deviations (τ_Se, τ_Sp) and the correlation ρ are reported. Moderator comparisons across AI technology, skin condition, study design, clinical setting, publication era, and comparator type remain descriptive because several subcategories contained too few studies for meta-regression.

## 3. Results

### 3.1. Study Selection and Inclusion

The systematic literature search identified a total of 30 studies meeting the predefined inclusion criteria for this review. Following database screening and full-text assessment, all 30 publications were retained for qualitative synthesis. Two articles were subsequently identified as formally retracted by their respective publishers, both as part of a large-scale mass retraction wave involving more than 500 papers across multiple Hindawi-owned open-access journals due to concerns regarding compromised peer review, potential paper mill involvement, and unverifiable data integrity (retraction notices are included in Supplementary Material S3). These two articles were excluded from all quantitative analyses and performance metric syntheses. Their citation information is preserved in the study characteristics table (Table 2) for transparency purposes. The final analytic cohort therefore comprised 28 non-retracted studies. Throughout this manuscript, the two retracted studies are marked with a dagger (†) in every table. They are included only in the descriptive, qualitative-synthesis totals reported as *n* = 30, while all quantitative pooled analyses, forest plots, and the Reitsma bivariate model are based on the 28 non-retracted studies (*n* = 28).

Included studies span a publication period of 22 years, from 2003 to 2025, reflecting the full trajectory of AI application in dermatology from early computational neural network experiments to contemporary large language model (LLM)- and vision–language model (VLM)-based tools. The distribution of publications was markedly skewed toward the most recent decade, with 25 of 30 studies (83.3%) published between 2020 and 2025, 3 studies (10.0%) published between 2018 and 2019, and 2 studies (6.7%) published before 2010.

### 3.2. Characteristics of Included Studies

The 30 included studies were published across a diverse range of high-impact peer-reviewed journals, including *The Lancet Digital Health*, *JAMA Network Open*, *JAMA Dermatology*, *Annals of Oncology*, *Nature Communications*, *British Journal of Dermatology*, *Journal of the American Academy of Dermatology*, *European Journal of Cancer*, *Journal of Investigative Dermatology*, *Journal of the European Academy of Dermatology and Venereology*, *PLoS One*, and several specialty digital health journals (*JMIR Dermatology*; *Scientific Reports*; *Journal of Proteomics*).

Study designs were heterogeneous (Table 3). The largest category was retrospective cohort and model development studies (*n* = 9; 30.0%), followed by prospective cohort studies and clinical trials (*n* = 8; 26.7%) and diagnostic accuracy/reader comparison studies (*n* = 6; 20.0%). Three studies (10.0%) were RCTs or prespecified analyses derived from RCT data, including the nested cost-effectiveness sub-study of Lindsay et al. [32], providing the highest level of clinical evidence available within the corpus. Cross-sectional and survey-based studies accounted for two publications (6.7%). Notably, only three RCT-level studies exist in the entire 22-year corpus [29,32,35], showing a persistent gap between the availability of AI diagnostic tools and their rigorous evaluation in interventional trial settings.

Sample sizes varied substantially across study types. Model development and reader studies typically involved hundreds of curated images rather than patient-level cohorts. The largest patient-level cohort was the Melanoma MEGA-study [33] encompassing 1653 patients from an international multicenter population. Other large prospective datasets included Winkler et al. [39] with 236 patients and 1075 clinically relevant melanocytic lesions (CRMLs), and another study with 309 high-risk patients followed over a 2-year RCT [32]. The smallest study was Gantenbein et al. [30], an AI-driven prevention pilot involving 60 healthy young women.

### 3.3. Geographic Distribution

Geographically, the included studies originated predominantly from European and Asia-Pacific settings (Table 4). European studies accounted for 14 publications (46.7%), with Germany contributing the largest national share (6 studies), followed by the Netherlands, Sweden, Switzerland, France, Spain, and Italy. Asia-Pacific and Oceanian studies comprised five publications (16.7%), including two from Australia, two from South Korea, and one from India. Three studies originated exclusively from China (10.0%), though this includes two retracted publications [40,44]. North American studies numbered three (10.0%), all from the United States. Five additional studies (16.7%) involved international multicenter collaborations spanning multiple continents, typically combining European and North American institutions.

The geographic concentration of AI dermatology research in Europe and East Asia shows both the presence of large academic dermatology centers with extensive dermoscopy archives and the regulatory environments in these regions that have facilitated CE-marking and clinical validation of AI medical devices. The relative underrepresentation of low- and middle-income country settings, with the exception of India, Marri et al., 2024 [25], is a limitation of the current evidence base, particularly given that access to specialist dermatology care remains severely constrained across large portions of sub-Saharan Africa, South Asia, and Latin America, where AI-enabled teledermatology could have the greatest transformative impact.

### 3.4. Dermatological Conditions Addressed

The distribution of targeted dermatological conditions was markedly skewed toward melanoma and melanocytic lesions (Table 5). Melanoma constituted the primary disease focus in 18 of 30 studies (60.0%), encompassing applications ranging from dermoscopic diagnosis and whole-body screening to digital pathology, lymph node staging, and precision oncology.

Beyond skin cancer, onychomycosis was the subject of two studies (6.7%): the retrospective EHR cohort of Roustan et al. [19] and the prospective comparative study of Kim et al. [42]. One study each addressed lupus erythematosus [27], acne vulgaris [35], and vitiligo [45]. Two studies evaluated AI across broad arrays of dermatoses, either through wide-spectrum consumer mobile apps (Marri et al. [25]) or through automated histopathological report generation covering multiple malignancy types (Tran et al. [24]).

This concentration of research effort creates a knowledge gap regarding AI performance across the far broader spectrum of inflammatory, infectious, and autoimmune skin conditions that constitute the majority of dermatological workload in clinical practice.

### 3.5. AI Technology and Temporal Evolution

The AI technologies employed across the included studies reflect the broader evolution of machine learning and computer vision over the last two decades, from early rule-based systems and classical artificial neural networks to contemporary foundation models (Table 6 and Table 7).

#### 3.5.1. Early Era: Classical Neural Networks (2003–2009)

The two earliest studies in this corpus [45,46] employed classical artificial neural networks (ANNs) operating on handcrafted feature inputs rather than raw image data. Hoffmann et al. [46] developed the DANAOS (diagnostic and neural analysis of skin cancer) expert system, an ANN trained on 2218 calibrated digital dermoscopic images collected prospectively from 13 dermatology centers in 9 European countries. DANAOS demonstrated performance similar to that of dermatologists as published in the literature, representing a landmark validation of computer-aided dermoscopy analysis and anticipating the concept. Cazzaniga et al. [45] applied discriminant and regression neural networks to clinical and photographic data from 325 vitiligo patches, achieving 66.5% accuracy in classifying excimer laser treatment responders from non-responders, representing the first application of AI to treatment response prediction in a pigmentary skin condition. These early studies were constrained by small datasets, handcrafted feature engineering, and lack of GPU-accelerated training, yet they established the conceptual foundations that subsequent deep learning approaches would operationalize at scale. Despite the proof of concept established by early computational dermoscopy studies, no studies meeting our eligibility criteria were identified in the period 2010 to 2017.

#### 3.5.2. Rise of Deep Learning: Convolutional Neural Networks (2018–2020)

The transition from classical ANNs to deep CNNs fundamentally transformed the field. CNNs eliminated the need for manual feature engineering and enabled end-to-end training on large image corpora. Three pivotal reader comparison studies published between 2018 and 2019 established the paradigm that CNN-based AI could match or surpass specialist dermatologists in melanoma recognition from dermoscopic images.

Haenssle et al. [22] reported that a modified Google Inception V4 CNN achieved an AUC of 0.95 for dermoscopic melanoma classification, outperforming 58 international dermatologists from 17 countries at both experience levels tested (Level I: dermoscopy only, AUC 0.79; Level II: dermoscopy plus clinical information, AUC 0.82). The CNN demonstrated higher sensitivity (95%) and specificity (80%) than both clinician groups, a finding that attracted widespread attention in the dermatological and broader medical AI communities. One year later, Brinker et al. [36] extended this paradigm using a CNN trained exclusively on 12,378 open-source dermoscopic images, demonstrating superior performance over 136 of 157 dermatologists (87%) from 12 German university hospitals across all seniority levels, with CNN sensitivity of 82.3% versus dermatologist sensitivity of 67.2%. Around the same time, Phillips et al. [28] independently validated comparable CNN superiority over board-certified dermatologists, reinforcing the robustness of these findings across different CNN architectures and testing datasets.

In 2020 Kim et al. [42] provided the first prospective evaluation of a DNN for onychomycosis diagnosis, finding that the algorithm (AUC 0.751) was statistically equivalent in performance to both dermoscopic examination (AUC 0.755) and experienced board-certified dermatologists (sensitivity 73.0%), with the additional practical advantage that AI analysis used standard clinical photographs obtainable by non-physicians rather than requiring specialist dermoscopic equipment.

#### 3.5.3. Clinical Validation and Real-World Translation (2021–2023)

Between 2021 and 2023, the field shifted from demonstrating that AI could match experts on curated testing sets toward evaluating clinical utility in real-world settings, validating regulatory-approved devices, and exploring histopathological applications. Höhn et al. [41] in 2021 combined CNN-based whole-slide-image classification with patient demographic data for histopathological melanoma–nevus differentiation, achieving an AUROC of 92.3% for the CNN alone and demonstrating that selective fusion with patient metadata improved balanced accuracy from 83.2% to 86.7% for low-confidence cases. Jansen et al. [31] extended CNN application to melanoma lymph node staging on hematoxylin and eosin-stained sentinel lymph node sections, achieving AUC > 0.96 on two independent test cohorts without requiring immunohistochemistry.

Sangers et al. [34] provided a clinically grounded prospective multicenter validation of a CE-marked AI mHealth application in 785 lesions from 372 patients in the Netherlands, reporting sensitivity of 86.9% and specificity of 70.4%, and identifying a significant performance disparity between iOS (91.0%) and Android (83.0%) platforms. Han et al. [29] published the first RCT demonstrating that AI-assisted diagnosis significantly improved non-expert physician accuracy for skin neoplasms (53.9% vs. 43.8% unaided; *p* = 0.019), providing evidence for the clinical decision support role of AI. Kommoss et al. [43] demonstrated that a market-approved CNN reduced incorrect management of diagnostically “unclear” face and scalp lesions from 58.8% to 4.1% for malignant cases. Menzies et al. [17] found that a seven-class mobile AI algorithm achieved diagnostic accuracy equivalent to specialist dermatologists (absolute difference +1.2%; 95% CI −6.9 to +9.2), while demonstrating that not all AI algorithms perform equivalently: the ISIC algorithm tested in the same setting was significantly inferior to specialists (Δ = −11.6%).

#### 3.5.4. Foundation Models, Multimodality, and Next-Generation AI (2024–2025)

The most recent stratum of studies reflects a new technological inflection in dermatological AI, characterized by multimodal architectures, foundation models, 3D-imaging integration, and AI applications beyond binary diagnosis. Papachristou et al. [18] demonstrated that a smartphone-based CNN decision support tool used prospectively by primary care physicians achieved AUROC 0.960 for melanoma, with 100% sensitivity for invasive melanoma (AUROC 0.988), establishing a compelling clinical case for AI-enhanced primary care referral pathways. Li Q et al. [27] deployed a multimodal deep learning system integrating clinical images, multicolor immunohistochemistry images, and structured clinical data to classify 13 skin conditions, including lupus erythematosus subtypes, achieving AUC 0.9844 and improving dermatologist diagnostic accuracy from 66.9% to 81.3% (*p* = 0.0004).

Tran et al. [24] introduced HistoGPT, the first vision–language foundation model for dermatopathology, trained on 15,129 WSIs from 6705 patients. HistoGPT generates free-text dermatopathology reports from gigapixel whole-slide images in a zero-shot fashion, matching human pathologist report quality for common homogeneous malignancies. Aung et al. [23] demonstrated that AI-driven quantification of tumor-infiltrating lymphocytes using the Hover-NeXt deep learning architecture outperformed traditional pathologist scoring in predicting immunotherapy response and survival in advanced melanoma. Guedes et al. [33] integrated proteogenomics, AI-driven digital pathology, and machine learning analytics in 1653 patients through the Melanoma MEGA-Study, identifying novel protein biomarker signatures for immune evasion and therapeutic resistance.

In the domain of prevention, Gantenbein et al. [30] deployed a generative AI-based facial photoaging simulation tool, finding that 91.7% of young women were motivated to reduce UV exposure immediately following the simulation, with 96% reporting persistent behavior modification at a 2-year follow-up.

### 3.6. Teledermatology and Primary Care AI

Three studies specifically evaluated AI in teledermatology or primary care contexts. Papachristou et al. [18] demonstrated that primary care physicians using an AI smartphone application achieved 100% sensitivity for invasive melanoma in a Swedish primary care setting. Le Lay et al. [38] validated an AI-assisted tele-expertise platform for common skin diseases in a French proof-of-concept study, achieving top-three diagnostic accuracy of 90% with significant improvement between algorithm versions 1 and 2. Marri et al. [25] evaluated the Aysa consumer AI application in an Indian semiurban setting, achieving top-three sensitivity of 86.1% across 12 dermatosis categories, but with notably poor performance for malignant tumors (top sensitivity 10%).

### 3.7. Treatment, Prevention, and Attitudinal Studies

Beyond diagnosis, three studies evaluated AI for treatment response prediction: Aung et al. [23] for immunotherapy response prediction via TIL quantification, Cazzaniga et al. [45] for excimer laser response prediction in vitiligo, and Guedes et al. [33] for precision oncology stratification in melanoma. Two studies evaluated AI-driven treatment recommendations: Roustan et al. [19] using NLP-based EHR analysis to characterize real-world outcomes in onychomycosis, and Ghazanfar et al. [35] demonstrating that an ML-based skincare recommendation system significantly outperformed self-selected treatment for mild-to-moderate acne vulgaris. Two attitudinal studies [20,26] examined patient and dermatologist preferences for AI integration in skin cancer diagnostics, finding that patients preferred augmented intelligence (AI + dermatologist) over either AI alone or unassisted dermatology, and that 83.4% of patients preferred AI-augmented consultations. Table 8 summarizes the above.

### 3.8. Diagnostic Performance of AI Systems

Reported diagnostic performance metrics varied substantially across studies (Table 9). Among CNN-based dermoscopy studies, reported AUC values ranged from 0.826 (classical ANN, Hoffmann et al. [46]) to 0.960 (smartphone CNN, Papachristou et al. [18]). In the pivotal comparative studies, Haenssle et al. [22] reported CNN AUC of 0.95 versus dermatologist AUC of 0.79–0.82; Brinker et al. [36] found CNN sensitivity of 82.3% and specificity of 77.9% versus dermatologist sensitivity of 67.2% and specificity of 62.2%. Several studies noted that AI performance degrades with image quality, lesion rarity, or mismatched population demographics (as demonstrated by Mitre et al. [21]).

The single RCT directly evaluating AI-assisted clinical decision support for skin neoplasm diagnosis, Han et al. [29], demonstrated a significant improvement in non-expert physician accuracy with AI assistance (53.9% vs. 43.8%; *p* = 0.019). The RCT by Ghazanfar et al. [35] extended this to treatment. Their ML-generated personalized skincare recommendations produced significantly greater acne severity reduction compared with self-selected treatment over 8 weeks. Lindsay et al. [32], while demonstrating clinical non-inferiority of 3D TBP-based AI surveillance, found that this approach was not cost-effective at current implementation costs, with 0% probability of cost-effectiveness from a government payer perspective at 2 years.

### 3.9. Forest Plot Analysis

The forest plots summarize diagnostic performance as AUROC. Pooling was done on the logit(AUROC) scale (random-effect REML; k = 9 independent testing sets), so Figure 2 displays logit values. The SPSS summary diamond is 2.48 (95% CI 1.91–3.06; I^2^ = 89%; τ^2^ = 0.60). Back-transformation with AUROC = 1/(1 + e^(−logit)^) gives a pooled AUROC of 0.92 (95% CI 0.87–0.96).

Several melanoma-focused studies reported strong discrimination. Haenssle et al. [22] obtained AUROC 0.95 on the independent test-set-300. Phillips et al. [28] reported an iPhone histology-verified AUROC of 0.901 (0.863–0.940), and Papachristou et al. [18] reported an overall AUROC of 0.960 (0.928–0.980). Hoffmann et al. [46] reported multicenter ROC area of 0.826 ± 0.021, and Jansen et al. [31] reported section-level AUROC values of 0.963 and 0.986 at two independent test centers.

Pooling of sensitivity was done on the logit scale in SPSS (random-effect REML; k = 7 independent 2 × 2 tables), so Figure 3 displays logit values. The SPSS summary diamond is 2.08 (95% CI 1.45–2.71; I^2^ = 89%; τ^2^ = 0.55). Back-transformation with sensitivity = 1/(1 + e^(−logit)^) gives a univariate pooled sensitivity of 0.89 (95% CI 0.81–0.94). The primary manuscript estimate is the Reitsma bivariate model, which jointly analyses sensitivity and specificity: 0.88 (95% CI 0.82–0.93). On the 0–1 scale, study sensitivities ranged from 0.70 (Kim et al. [42]) to 0.95 (Haenssle et al. [22]; Jansen et al. [31] Center 2). Papachristou et al. [18] contributed the overall Youden point (20/21, 0.95), not the invasive-only value of 1.00, which is the same 253 lesions.

Some variability was observed due to differences in study design, populations, imaging modalities, and AI architectures. Technologies ranged from traditional neural networks [46] and CNNs [22,36] to smartphone-based applications [18] and multimodal systems [27] across conditions including melanoma, skin neoplasms, onychomycosis, and lupus erythematosus. Given this substantial heterogeneity (I^2^ = 89–97% across the three pooled outcomes), the pooled AUROC, sensitivity, and specificity estimates should be interpreted with caution: they summarize a highly diverse set of AI technologies, patient populations, and clinical settings, and should not be read as a single, precise, generalisable performance figure applicable to any individual clinical context.

Pooling of specificity was done on the logit scale in SPSS (random-effect REML; k = 7 independent 2 × 2 tables), so Figure 4 displays logit values. The SPSS summary diamond is 1.74 (95% CI 1.08–2.40; I^2^ = 97%; τ^2^ = 0.75). Back-transformation with specificity = 1/(1 + e^(−logit)^) gives a univariate pooled specificity of 0.85 (95% CI 0.75–0.92). The primary manuscript estimate is the Reitsma bivariate model: 0.85 (95% CI 0.75–0.91).

On the 0–1 scale, study specificities ranged from 0.70 (Sangers et al. [34]) to 0.96 (Jansen et al. [31] Center 2). Papachristou et al. [18] contributed the overall Youden specificity of 0.85, not the invasive-only 0.93.

### 3.10. Data Quality, Risk of Bias, and Retracted Studies

Risk of bias was assessed qualitatively across included studies. First, the majority of dermoscopy-based AI studies used retrospective, curated image datasets with balanced class distributions that are unrepresentative of real-world clinical prevalence. Melanoma prevalence in real-world dermatology practice is typically 2–5% of assessed lesions [47,48,49]. Most CNN evaluation sets used melanoma prevalence of 30–50%, substantially inflating sensitivity estimates and potentially reducing the positive predictive value achievable in clinical deployment.

Second, geographic and demographic homogeneity in training datasets represents a significant source of systematic bias. The ISIC dataset contains a disproportionate representation of Fitzpatrick phototype I–III skin [50,51] from European and Australian populations, with minimal representation of Fitzpatrick phototype V–VI skin. Mitre et al. [21] provide direct empirical evidence of the clinical harm this bias produces, reporting near-zero AI specificity (0–4.3%) for benign acral lesions in Black patients at a standard 95% sensitivity threshold.

Third, comparator standardization was inconsistent across reader studies. Dermatologist comparators ranged from single junior clinicians to panels of 58 international experts. The conditions of clinical information available to human readers varied, and the definition of “gold standard” ranged from histopathological diagnosis to clinical consensus, introducing systematic heterogeneity in relative performance estimates.

The two retracted studies involved claims of AI-based image analysis in dermatological contexts (3D skin CT in pediatric psoriasis [40]; deep learning melanoma detection from histopathological slides [44]) that could not be independently verified following retraction.

## 4. Discussion

This meta-analysis demonstrates a pooled AUROC of 0.92 (95% CI 0.87–0.96) for AI diagnostic discrimination, with Reitsma sensitivity 0.88 (0.82–0.93) and specificity 0.85 (0.75–0.91), consistently matching or surpassing specialist dermatologist performance in direct comparisons. We found similar evidence in the literature [52,53]. These findings align closely with those of Salinas et al. [54], whose meta-analysis of 30 studies reported a pooled AUC of 0.91 (95% CI 0.89–0.93) for AI versus clinicians in skin cancer diagnosis, with AI performing equivalently to or better than dermatologists in the majority of included studies. The most recent prospective evidence synthesis by Laiouar-Pedari et al. [55] confirmed that AI-assisted decision support reduced melanoma miss rates compared with unaided clinicians.

A recurring theme in our results is the gap between controlled benchmark performance and real-world deployment. Heinlein et al. [56] prospectively demonstrated in a 29-center German study that AI assistance improved melanoma recognition in patient care, though performance gains were smaller than those reported in retrospective reader studies. Thomas et al. [57] similarly documented real-world performance degradation relative to pre-deployment validation in a post-market surveillance analysis. The CLEAR Derm consensus guidelines provide a standardised checklist for evaluating the rigor of image-based AI reports [58]. This broader principle of context-dependent clinical decision-making is also supported by real-world evidence from other medical domains, where patient comorbidities, baseline risk, and treatment characteristics can substantially influence clinical outcomes and therefore need to be considered when translating evidence into individualized care [59].

The near-zero specificity (0–4.3%) observed by Mitre et al. [21] for benign acral lesions in Black patients is the most clinically urgent finding in the present review, and is not an isolated observation. Daneshjou et al. [60] demonstrated significantly worse AI performance across darker Fitzpatrick phototypes on a diverse curated dataset, while Guo et al. [61] documented systematic underrepresentation of non-White skin types in published AI training datasets. Dovigi et al. [62] further reviewed bias, ethics, and regulatory considerations in dermatological AI, concluding that existing regulatory pathways fail to mandate demographic subgroup performance reporting as a condition of market approval.

Our results reveal a pronounced concentration of AI research on melanoma (60% of included studies), with comparatively sparse evidence for inflammatory and infectious dermatoses. This mirrors the evidence gap identified by Cai et al. [63] in a systematic review of AI for inflammatory skin disease severity assessment, which found high heterogeneity and modest performance for conditions such as psoriasis and atopic dermatitis. For acne, CNN-based grading systems have shown promise [64], while machine learning models have demonstrated early utility in predicting atopic dermatitis flares from patient-reported and sensor data [65]. The AI teledermatology literature confirms that algorithms embedded in primary care workflows can improve early detection rates, particularly in geographically underserved populations where specialist access is limited [63].

Beyond image interpretation alone, artificial intelligence is increasingly driving the transition toward personalized medicine through the integration of multimodal clinical, molecular, and microbiome-derived data, enabling more individualized diagnostic, prognostic, and therapeutic strategies [65]. Hyperspectral imaging combined with machine learning is a further emerging modality with reported potential to enhance skin cancer classification beyond conventional RGB dermoscopy. This technology was not represented among the 30 studies included in the present corpus and does not currently map onto the AI technology taxonomy used in Table 6, representing a direction for future updates of this review [66]. The convergence of artificial intelligence with smart biomaterials and connected technologies is also expanding beyond diagnostic support into medical traceability and forensic medicine. Intelligent materials incorporating near-field communication technology have demonstrated feasibility for durable identification [67].

Health economic considerations remain a critical, but underdeveloped domain. Our paper contains only one formal cost-effectiveness analysis [32], which found AI-based total-body photography surveillance not cost-effective over a 2-year horizon. This is consistent with a broader modeling study by Gomez Rossi et al. [68], which found that AI-assisted melanoma detection reached cost-effectiveness thresholds only under specific prevalence and sensitivity assumptions. Regulatory oversight of AI dermatology applications remains fragmented. Wongvibulsin et al. [69] found that the majority of commercial dermatology apps with AI features available in app stores lacked regulatory clearance, a concern also raised by Matin and Dinnes [70] regarding smartphone-based risk assessment tools.

In computational pathology, AI systems for dermatopathology demonstrate consistently high accuracy for melanoma classification on whole slide images, as confirmed by Lalmalani et al. [71]. A Cochrane review by Ferrante di Ruffano et al. [72] identified a high risk of bias in early computer-assisted dermoscopy studies due to methodological deficiencies in patient selection and reference standard definition.

Furthermore, the absence of prospective, registry-level outcome data beyond short-term diagnostic accuracy metrics limits conclusions regarding the impact of AI on patient survival and long-term clinical outcomes. In addition, risk-of-bias appraisal in this review was qualitative rather than based on a formal domain-by-domain QUADAS-2/RoB 2 scoring system with a quantitative inter-rater agreement statistic. A structured, study-level risk-of-bias table is identified as a priority for a future update of this review.

Looking forward, multimodal foundation models such as PanDerm (Yan et al. [73], trained across diverse dermatological image types, represent the next generation of broadly applicable AI in dermatology, while SkinGPT-4 (Zhou et al. [74]) demonstrates that pretrained multimodal large language models can support differential diagnosis and management reasoning from clinical images.

## 5. Conclusions

This meta-analysis provides the most temporally comprehensive synthesis to date of artificial intelligence applications across dermatology. The evidence base demonstrates a consistent and reproducible trajectory: from early classical neural networks achieving dermatologist-equivalent performance on curated image sets, through the deep learning revolution catalyzed by landmark CNN studies, to the current generation of foundation models, multimodal systems, and AI-integrated clinical workflows spanning diagnosis, pathology, screening, and precision oncology.

Across diagnostic applications, the pooled AUROC was 0.92 (95% CI 0.87–0.96), with Reitsma sensitivity 0.88 (0.82–0.93) and specificity 0.85 (0.75–0.91), frequently matching or surpassing specialist dermatologist performance. Randomized controlled trial evidence confirms that AI decision support significantly improves non-expert diagnostic accuracy, establishing a robust evidentiary basis for AI as a clinical augmentation tool rather than a standalone replacement. In histopathological applications, deep learning systems have demonstrated high accuracy for melanoma detection in whole slide images, lymph node staging, and tumor-infiltrating lymphocyte quantification.

However, critical limitations temper these findings. Algorithmic racial bias represents an urgent patient safety concern requiring immediate attention in dataset curation and regulatory review. Health economic analysis reveals that clinical accuracy alone does not guarantee cost-effectiveness. Furthermore, the absence of prospective, registry-level outcome data beyond short-term diagnostic accuracy metrics limits conclusions regarding the impact of AI on patient survival and long-term clinical outcomes.

In conclusion, AI in dermatology has transitioned from proof of concept to clinically validated reality across multiple application domains. Responsible translation into routine practice requires technology-specific validation, equitable dataset representation, rigorous health economic evaluation, and structured post-market surveillance.

## Figures and Tables

**Table 1:** Predefined inclusion and exclusion criteria.

Criterion Type	Criteria
Inclusion	1. Original research studies (clinical trials, cohort studies, case-control, cross-sectional, RCTs)
	2. Studies evaluating AI, machine learning, deep learning, neural networks, or related computational methods applied to any dermatological condition or outcome
	3. Studies reporting at least one quantitative AI performance metric (AUC, sensitivity, specificity, accuracy, F1 score, diagnostic odds ratio) OR a clinical outcome measure (accuracy change, management impact, cost-effectiveness)
	4. Human skin diseases as the primary subject (including oncological, infectious, inflammatory, pigmentary, and autoimmune conditions)
	5. Studies involving any clinical setting (primary care, secondary/tertiary care, teledermatology, pathology laboratory)
	6. Full-text articles published in peer-reviewed journals
	7. English-language publications
Exclusion	1. Review articles, systematic reviews, meta-analyses, editorials, letters, commentaries, conference abstracts without full-text data
	2. Studies focused exclusively on non-dermatological conditions (e.g., retinal imaging, radiology, cardiology AI)
	3. Animal studies or in vitro studies without human skin data
	4. Studies not reporting any quantitative performance metric or clinical outcome
	5. Duplicate publications reporting identical datasets without new analyses
	6. Studies with unavailable full text after library access attempts
	7. Retracted publications (identified through Retraction Watch database and PubMed retraction notices)
	8. AI applications for administrative, scheduling, or non-clinical dermatology purposes

AI = artificial intelligence; AUC = area under the ROC curve.

**Table 2:** Characteristics of included studies.

Study	Journal	Location	Study Design	Sample Size	Skin Condition	AI Application
Menzies et al., 2023 [17]	Lancet Digit Health	Australia	Prospective multicenter diagnostic trial	127 pts; 172 lesions	Pigmented skin cancer (7 classes)	Diagnosis/classification
Papachristou et al., 2024 [18]	Br J Dermatol	Sweden	Prospective multicenter clinical trial	228 pts; 253 lesions	Cutaneous melanoma	Diagnosis/screening/teledermatology
Roustan et al., 2025 [19]	Sci Rep	Spain	Retrospective EHR cohort (NLP)	408 pts	Onychomycosis	Treatment recommendation/EHR mining
Haggenmüller et al., 2024 [20]	J Am Acad Dermatol	Germany	Prospective multicentric survey	178 patients and 115 dermatologists	Skin cancer (melanoma)	Patient/clinician attitudes
Mitre et al., 2024 [21]	J Am Acad Dermatol	USA	Multicenter observational	249 lesions; 100 Black pts	Acral pigmented lesions/melanoma	Diagnosis—equity/bias assessment
Haenssle et al., 2018 [22]	Ann Oncol	Germany	Retrospective reader study	300 images; 58 dermatologists	Melanoma (dermoscopy)	Diagnosis/classification
Aung et al., 2025 [23]	JAMA Netw Open	USA/Sweden	Multicenter retrospective cohort	Melanoma pts treated 2016–2023	Melanoma (advanced; ICI treatment)	Pathology/treatment response
Tran et al., 2025 [24]	Nat Commun	Germany/USA/Netherlands	Model dev. + multicenter validation	15,129 WSIs; 6705 pts	Multiple skin malignancies (WSI)	Pathology—automated report generation
Marri et al., 2024 [25]	JMIR Dermatol	India	Cross-sectional diagnostic accuracy	N NR; 12 disease categories	Broad dermatoses (12 classes)	Diagnosis/teledermatology
Goessinger et al., 2024 [26]	J Eur Acad Dermatol	Austria/Switzerland	Prospective observational	201 pts; 205 consultations	Melanoma (TBP screening)	Screening; clinician/patient attitudes
Li Q et al., 2024 [27]	J Eur Acad Dermatol	China (25 sites)	Multicenter retrospective diagnostic	446 cases; 800+ images	Lupus erythematosus (13 conditions)	Diagnosis—multimodal AI
Phillips et al., 2019 [28]	JAMA Netw Open	USA	Retrospective diagnostic accuracy	Large dermoscopy image set	Melanoma (dermoscopy)	Diagnosis/screening
Han et al., 2022 [29]	J Invest Dermatol	South Korea	Single-center RCT	576 cases (Fitzpatrick III–IV)	Skin neoplasms	AI-assisted diagnosis (non-experts)
Gantenbein et al., 2025 [30]	Dermatology	Switzerland	Prospective observational pilot	60 participants; 25 at 2-yr FU	Skin cancer prevention (melanoma)	Patient education/prevention
Jansen et al., 2023 [31]	Eur J Cancer	Germany	Retrospective multicenter model dev.	SLN histology sections (N NR)	Melanoma (lymph node staging)	Pathology/AI-assisted staging
Lindsay et al., 2025 [32]	JAMA Dermatol	Australia	CEA from RCT	309 high-risk pts (2-yr trial)	Melanoma (high-risk screening)	Screening—cost-effectiveness
Guedes et al., 2025 [33]	J Proteomics	International	Multicenter retrospective cohort	1653 pts (primary + mets)	Melanoma (precision oncology)	Pathology/multiomics/AI analytics
Sangers et al., 2022 [34]	Dermatology	Netherlands	Prospective multicenter	785 lesions; 372 pts	Premalignant/malignant lesions	Screening/mHealth validation
Ghazanfar et al., 2025 [35]	JMIR Dermatol	Denmark	8-week evaluator-blinded RCT	N NR (mild–mod. acne)	Acne vulgaris	Treatment recommendation (ML)
Brinker et al., 2019 [36]	Eur J Cancer	Germany (12 sites)	Retrospective reader study	100 images; 157 dermatologists	Melanoma (dermoscopy)	Diagnosis/classification
Marchetti et al., 2023 [37]	J Eur Acad Dermatol	USA	Retrospective observational pilot	35 pts; 23,538 lesions identified	Melanoma (whole-body screening)	Screening/AI-assisted detection
Le Lay et al., 2024 [38]	Eur J Dermatol	France	Proof-of-concept diagnostic accuracy	70 participants; 195 photographs	Common skin diseases	Teledermatology/AI triage
Winkler et al., 2024 [39]	Eur J Cancer	Europe	Prospective multicenter	236 pts; 1075 CRMLs	Melanocytic lesions (screening)	Screening—TBP/ATBM algorithm
Wang et al., 2022 cited by Li Pomi et al., 2024 [40] †	Comput Math Methods Med	China	Retrospective (cross-sectional)	Paediatric psoriasis (N NR)	Psoriasis (paediatric)	Diagnosis/3D CT imaging
Höhn et al., 2021 [41]	Eur J Cancer	Germany	Retrospective model dev.	431 WSIs (2 labs)	Melanoma vs. nevus	Pathology/WSI classification
Kim et al., 2020 [42]	PLoS One	South Korea	Prospective observational	90 pts (Sep 2018–Jul 2019)	Onychomycosis	Diagnosis/classification
Kommoss et al., 2023 [43]	Eur J Cancer	Germany	Retrospective reader study	100 FSLs; 64 dermatologists	Face/scalp lesions (unclear cases)	AI decision support (unclear lesions)
Li T et al., 2021 [44] †	J Healthc Eng	China	Retrospective multicenter	Melanoma histo slides	Melanoma (histopathology)	Pathology/diagnosis
Cazzaniga et al., 2009 [45]	Dermatology	Italy	Secondary analysis of RCT data	325 vitiligo patches	Vitiligo (excimer laser)	Treatment response prediction
Hoffmann et al., 2003 [46]	Br J Dermatol	Europe (9 countries)	Multicenter prospective	2218 dermoscopic images	Pigmented skin lesions/melanoma	Diagnosis/classification (early NN)

† Wang et al. (doi: 10.1155/2023/9841460)—retracted study cited by Li Pomi et al. [40] excluded from all quantitative pooling (retraction notice in Supplementary Material S3); Li T et al. (doi: 10.1155/2021/5972962) retracted following an investigation led by Wiley and Hindawi that let to a discovery of 1200 articles with compromised peer review (retraction notice in Supplementary Material S3) [44]. Abbreviations: CEA = cost-effectiveness analysis; CRML = clinically relevant melanocytic lesion; EHR = electronic health record; FU = follow-up; NLP = natural language processing; NR = not reported; pts = patients; RCT = randomized controlled trial; SLN = sentinel lymph node; TBP = total body photography; WSI = whole slide image.

**Table 3:** Distribution of included studies by study design.

Study Design	Study Numbers	Proportion	Citation Numbers
Randomized controlled trial	3	10.0%	[29,32,35] ^1^
Prospective cohort/clinical trial	8	26.7%	[17,18,21,26,30,34,39,42]
Retrospective cohort/model development	9	30.0%	[19,23,24,27,31,33,38,41,45]
Diagnostic accuracy	6	20.0%	[22,28,36,37,43,46]
Cross-sectional study	2	6.7%	[20,25]
Retracted	2	6.7%	[40,44]
Total	30	100%	—

^1^ Ref. [32] is a randomized controlled trial with a nested cost-effectiveness sub-study; it is counted once, under RCT, to keep the total at 30.

**Table 4:** Geographic distribution of included studies.

Geographic Region	Study No.	Proportion (%)	Countries Represented
Europe	14	46.7%	Germany (6), Sweden (1), Netherlands (1), Switzerland (1), France (1), Spain (1), Italy (1), International/European consortium (2)
Asia-Pacific/Oceania	5	16.7%	Australia (2), South Korea (2), India (1)
East Asia	3	10.0%	China (3)—including 2 retracted
North America	3	10.0%	USA (3)
International/multicenter (multiple regions)	5	16.7%	USA + Europe + other (3); Sweden + intl. (1); Intl. Europe + USA (1)
Total	30	100%	—

**Table 5:** Distribution of included studies by targeted skin condition.

Skin Condition/Disease Focus	Studies No.	Proportion (%)	Citation Numbers
Melanoma (primary focus)	18	60.0%	[17,18,20,21,22,23,26,28,30,31,32,33,36,37,39,43,44,46] †
General skin cancer/multiple neoplasms	4	13.3%	[29,34,38,41]
Broad dermatoses/dermatopathology (multi-class)	2	6.7%	[24,25]
Onychomycosis	2	6.7%	[19,42]
Lupus erythematosus (subtypes + mimics)	1	3.3%	[27]
Acne vulgaris	1	3.3%	[35]
Vitiligo	1	3.3%	[45]
Psoriasis (pediatric—retracted)	1	3.3%	[40] †
Total	30	100%	—

† Retracted Wang et al. [40] and Li et al. [44].

**Table 6:** Classification of included studies by AI technology category.

AI Technology Category	Studies No.	Proportion (%)	Year Range	Citation Numbers
CNN/deep learning (dermoscopy/clinical image classification)	11	36.7	2018–2024	[17,21,22,28,29,36,37,38,40,42,43] †
Deep learning histopathology/whole slide image (WSI) analysis	3	10	2021–2023	[31,41,44] †
Mobile app/mHealth AI (CNN-based, consumer-facing)	3	10	2022–2024	[18,25,34]
3D Total-body photography + automated AI	3	10	2023–2025	[26,32,39]
Multimodal deep learning (multi-input data fusion)	2	6.7	2024–2025	[23,27]
NLP/machine learning (EHR mining/recommendation engine)	2	6.7	2025	[19,35]
Classical artificial neural network (pre-deep learning)	2	6.7	2003–2009	[45,46]
LLM/VLM	1	3.3	2025	[30]
Generative AI/photoaging simulation	1	3.3	2025	[33]
AI multiomics/digital pathology integration	1	3.3	2024	[20]
Total	30	100	2003–2025	—

Total = 30 (including 2 retracted). † RetractedWang et al. [40] and Li et al. [44]. CNN = convolutional neural network; EHR = electronic health record; LLM = large language model; NLP = natural language processing; VLM = vision–language model; WSI = whole slide image.

**Table 7:** Temporal evolution of AI technologies in dermatology.

Era/Period	Years	Predominant AI Technology	Studies No.	Representative Citations
Early AI (pre-deep learning)	2003–2009	Classical artificial neural networks (ANN); rule-based expert systems; computer-aided diagnosis (CAD) using handcrafted dermoscopic features	2	Hoffmann et al. [46]; Cazzaniga et al. [45]
Rise of deep learning	2018–2019	Convolutional neural networks (CNN); deep learning for image classification; reader comparison studies establishing CNN superiority over clinicians	3	Haenssle et al. [22]; Phillips et al. [28]; Brinker et al. [36]
Clinical validation era	2020–2022	CNN-based dermoscopy AI in prospective clinical settings; mHealth apps (CE-marked); whole slide image (WSI) deep learning; RCT-level evidence	4	Han et al. [29]; Sangers et al. [34]; Höhn et al. [41]; Kim et al. [42]
Translation and real-world deployment	2023–2024	3D total body photography (TBP) + AI; AI-assisted teledermatology; multimodal deep learning (multi-input); real-world clinical trials; equity/bias evaluation	12	Menzies et al. [17]; Papachristou et al. [18]; Mitre et al. [21]; Haggenmüller et al. [20]; Li et al. [27]; Goessinger et al. [26]; Le Lay et al. [38]; Winkler et al. [39]; Marri et al. [25]; Marchetti et al. [37];] Kommoss et al. [43]; Jansen et al. [31];
Foundation models and precision AI	2025	Large language models (LLM); vision–language models (VLM); generative AI; AI multiomics integration; LLM-based automated pathology report generation	7	Roustan et al. [19]; Tran et al. [24]; Aung et al. [23]; Gantenbein et al. [30]; Ghazanfar et al. [35]; Guedes et al. [33]; Lindsay et al. [32]

**Table 8:** AI application categories across included studies.

Application Category	Study No. (%)	AI Technology Used	Conditions Addressed	Citation Numbers
Diagnosis/classification	18 (60%)	CNN-based dermoscopy; multimodal DL; mobile apps; deep neural networks	Melanoma, onychomycosis, lupus, skin neoplasms, broad dermatoses	[17,18,21,22,25,27,28,29,34,36,37,38,39,40,42,43,45,46] †
Pathology/histology	6 (20%)	WSI deep learning; VLM (HistoGPT); CNN-based histopathology; TIL quantification; multiomics	Melanoma, BCC, SCC, AK, mixed malignancies	[23,24,31,33,41,44] †
Screening/surveillance	5 (17%)	3D TBP + automated AI; mHealth apps; ATBM systems; sequential dermoscopy	Melanoma (high-risk), melanocytic lesions, general suspicious lesions	[18,26,32,37,39]
Treatment response prediction	3 (10%)	Multimodal DL; AI multiomics; classical NN	Advanced melanoma (ICI), vitiligo (laser), melanoma (precision oncology)	[23,33,45]
Treatment recommendation	2 (7%)	NLP/ML on EHR; ML-based recommendation engine	Onychomycosis, acne vulgaris	[19,35]
Patient education/prevention	2 (7%)	Generative AI (photoaging simulation); consumer-facing mobile AI app	Skin cancer prevention (melanoma), broad dermatoses	[25,30]
Teledermatology/remote triage	3 (10%)	AI-assisted tele-expertise platforms; smartphone apps in primary care	Common skin diseases, melanoma, suspicious lesions	[18,34,38]
Cost-effectiveness analysis	1 (3%)	3D TBP + AI-assisted surveillance (economic modeling)	Melanoma (high-risk)	[32]
Patient/clinician attitudes	2 (7%)	Survey-based; observational preference assessment	Skin cancer (AI-driven diagnostics)	[20,26]

† Retracted—Wang et al. [40] and Li et al. [44].

**Table 9:** Reported diagnostic performance metrics across included studies.

Study	Skin Condition	AI Technology	Study Design	AI Performance	Comparator Performance	Key Finding
Haenssle, 2018 [6]	Melanoma dermoscopy	Inception V4 CNN	Cross-sectional reader study	AUC 0.95; Sensitivity 95%; Specificity 80%	Dermatologists Level I: AUC 0.79; Level II: AUC 0.82	CNN outperformed majority of 58 international dermatologists
Brinker, 2019 [20]	Melanoma dermoscopy	Open-source CNN	Retrospective reader study	Sensitivity 82.3%; Specificity 77.9%	Dermatologists: Sensitivity 67.2%; Specificity 62.2%	CNN outperformed 136/157 (87%) dermatologists
Phillips, 2019 [12]	Melanoma (dermoscopy/clinical)	Deep learning CNN	Retrospective diagnostic accuracy	High accuracy; performance ≥ board-certified dermatologists	Multiple dermatologists (board-certified)	AI accuracy comparable to or exceeding specialist level
Kim et al. [42]	Onychomycosis	DNN	Prospective comparative	AUC 0.751; sensitivity 70.2%; specificity 72.7%	Dermoscopy AUC 0.755; dermatologists sensitivity 73.0%	DNN comparable to dermoscopy and specialists; no significant difference
Höhn et al. [41]	Melanoma vs. nevus (WSI)	CNN (histopathology)	Retrospective model dev.	AUROC 92.30%; balanced accuracy 83.17%	Patient-data classifier: balanced accuracy 83.42%	Naive fusion with patient data improved accuracy to 86.72%
Han et al. [29]	Skin neoplasms (mixed)	CNN-based multiclass AI	RCT	AI-assisted: 53.9% accuracy	Unaided: 43.8% accuracy	Significant improvement with AI assistance (*p* = 0.019); first RCT-level evidence
Sangers et al. [34]	Premalignant/malignant lesions	CE-marked mHealth app (CNN)	Prospective multicenter	Sensitivity 86.9%; specificity 70.4%	Histopathological ground truth	iOS sensitivity (91.0%) > Android (83.0%); real-world mHealth validation
Marchetti et al. [37]	Melanoma (3D whole-body)	Automated image processing + LR	Retrospective pilot	AUC 0.94; 75% reduction in lesions for review at 95% sensitivity	Null model/clinical examination standard	Substantial reduction in clinical workload demonstrated
Jansen et al. [31]	Melanoma LN metastases (WSI)	CNN (histopathology)	Retrospective multicenter	AUC 0.9630 (cohort 1); AUC 0.9856 (cohort 2)	Pathologist classification standard	High accuracy for metastasis detection without IHC
Kommoss et al. [43]	Face/scalp unclear lesions	CNN (Moleanalyzer-Pro^®^)	Retrospective reader study	Incorrect management decreased from 58.8% to 4.1% (malignant unclear cases) with CNN	Dermatologist diagnoses without AI	CNN most valuable precisely for diagnostically challenging cases
Winkler et al. [39]	Melanocytic lesions (TBP)	ATBM system (2D TBP + algorithm)	Prospective multicenter	92.9% CRML detection (baseline); 96.7% (follow-up); 100% new/changed lesions	Dermatologist-identified lesions as reference	All new/changed lesions detected; average exam time 14.1 min
Menzies et al. [17]	Pigmented skin cancer (7 classes)	7-class CNN (mobile)	Prospective multicenter trial	7-class AI ≡ specialists (Δ = +1.2%, 95% CI −6.9–9.2); ISIC AI inferior to specialists (Δ = −11.6%)	Specialist dermatologists and novice clinicians	Algorithm design critically determines real-world clinical performance
Papachristou et al. [18]	Cutaneous melanoma (primary care)	Smartphone CNN app	Prospective clinical trial	AUROC 0.960; sensitivity 95.2%; specificity 84.5%; invasive melanoma: AUROC 0.988; sensitivity 100%	Histopathological diagnosis (ground truth)	100% sensitivity for invasive melanoma in primary care setting
Mitre et al. [21]	Acral pigmented lesions (Black pts)	ISIC 2020 winner CNN	Multicenter observational	Specificity 4.3% (volar); 1.4% (dorsal); 0% (nail) at 95% sensitivity	Published performance in predominantly White cohort (specificity 37.4%)	Critical algorithmic bias; near-zero specificity in under-represented population
Li et al. [27]	Lupus erythematosus (13 conditions)	MMDLS	Multicenter retrospective	AUC 0.9844; sensitivity 0.8288; specificity 0.9852	Unaided dermatologist accuracy: 66.88% ± 6.94%	AI assistance improved dermatologist accuracy from 67% to 81% (*p* = 0.0004)
Le Lay et al. [38]	Common skin diseases	AI tele-expertise platform V2	Proof-of-concept	Top-3 sensitivity 88%; specificity 90%; top-3 accuracy 0.90 (0.83–0.97)	V1 algorithm (earlier version)	Significant performance improvement between algorithm versions
Cazzaniga et al. [45]	Vitiligo (excimer laser response)	Classical ANN	Secondary analysis RCT data	Discriminant NN accuracy 66.46%; Regression NN MAE 19.58	No strong single clinical predictor available	Early proof of concept: NN provides useful treatment response prediction
Hoffmann et al. [46]	Pigmented skin lesions/melanoma	Classical ANN (DANAOS)	Multicenter prospective	Multicenter ROC area 0.826 ± 0.021	Dermatologist performance from published literature	Landmark early multicenter European AI dermoscopy validation study

Abbreviations: AUC = area under the ROC curve; AUROC = area under the receiver operating characteristic curve; CRML = clinically relevant melanocytic lesion; DNN = deep neural network; LN = lymph node; LR = logistic regression; MMDLS = multimodal deep learning system; TBP = total-body photography; WSI = whole slide image.

## Data Availability

No new data were created or analyzed in this study. Data sharing is not applicable to this article.

## Supplementary Materials

The following supporting information can be downloaded at: https://www.mdpi.com/article/10.3390/diagnostics16172797/s1. Supplementary Material S1: Complete literature search results and study database. Supplementary Material S2: Data underlying the diagnostic accuracy analyses. Supplementary Material S3: PRISMA_checklist.
